# The Rate of Decline in Small Fibre Function Assessed Using Axon Reflex-Mediated Neurogenic Vasodilatation and the Importance of Age Related Centile Values to Improve the Detection of Clinical Neuropathy

**DOI:** 10.1371/journal.pone.0069920

**Published:** 2013-07-25

**Authors:** Prashanth R. J. Vas, Gerry Rayman

**Affiliations:** The Diabetes Research Centre, Ipswich Hospital NHS Trust, Suffolk, United Kingdom; St. Joseph's Hospital and Medical Center, United States of America

## Abstract

**Background:**

The LDIflare technique (LDIflare) is a simple non-invasive test of small fibre function in dorsal foot skin involving skin heating and measuring the size of the resulting axon reflex-mediated vasodilator (flare) response using a laser Doppler imager (LDI). This study establishes age-related normative reference ranges for the test and determines the rate of decline in small fibre function per decade. Additionally, the potential value of using age related centiles rather than Receiver Operator Curves (ROC) was explored by comparison of the sensitivity and specificity of each analytic technique in identifying clinical neuropathy.

**Methods:**

LDIflare areas were assessed in 94 healthy controls and 66 individuals with diabetes with (DN+, n = 31) and without clinical neuropathy (DN-, n = 35); neuropathy defined as a Neuropathy Disability Score ≥3. The age specific 5th centile values were used as the ‘cut-offs’ for the diagnosis of neuropathy from which sensitivity and specificity were calculated.

**Results:**

There was a significant age dependant decrease in LDIflare size (r = −0.42, p<0.0001) with no significant gender differences. The LDIflare size reduced 0.56 cm^2^ per decade which gives a percentage reduction of approximately 5.5% per decade. Using the normative 5th centiles as the cut-offs, the technique had a sensitivity of 77%, specificity of 90%, positive predictive value of 82% and negative predictive value of 87%.The ROC analysis gave a threshold of <3.66 cm^2^ for the cut-off, resulting in a sensitivity of 75%, specificity of 85%, positive predictive value of 74% and negative predictive value of 86%.

**Conclusions:**

There is an age dependent decrease in small fibre function in the foot of 5.5% per decade. Both analytic techniques demonstrate good sensitivity and specificity for detecting clinical neuropathy but the technique based on age centiles offers better diagnostic accuracy and is therefore proposed as the method of choice.

## Introduction

Recent publications suggest that damage to small nerve fibres may be the earliest feature of diabetic neuropathy, preceding large fibre involvement [1], [2], [3], [4]; however, assessing small fibre structure and function remains a challenge particularly if this is to be achieved non-invasively. Computerised quantitative sensory tests (of heat and cold pain and thermal thresholds are commonly used non-invasive tests but these are limited by their subjective nature [5], [6], [7]. Indeed, a previous review concludes that there is considerable variability in the reliability of each of these thermal quantitative sensory test parameters [6]. Skin biopsy with measurement of intra-epidermal nerve fibre density (IENFD) is an excellent tool; however it is invasive limiting its use in prospective studies. Measurement of corneal nerve fibre density by corneal confocal microscopy shows great promise [8]. It has been shown to have good correlation with intraepidermal nerve fibre density. However, both IENFD and corneal confocal microscopy assess structure rather than function and importantly, the latter does not test the affected region directly. Nerve conduction velocity and amplitude studies, are the gold standard clinical tests for diagnosing neuropathy, frequently used in clinical practice as well as endpoints in neuropathy trials. However, these primarily assess large fibres and so cannot be used to exclude small fibre neuropathy in conditions where the neuropathy is limited to small fibres alone.

The LDIflare technique is a novel, non-invasive and objective method for assessing small fibre function in the dorsal foot skin [3]. It involves foot skin heating and measurement of the area of the resulting axon reflex mediated vasodilatation using laser Doppler imagery. This area we have termed the LDIflare. Though a test of function it correlates with the structural measures of both dermal nerve fibre density and IENFD [9], [10]. It is invariably abnormal in those with diabetic neuropathy. Furthermore, it is reduced in early type 2 diabetes and impaired glucose tolerance when quantitative sensory tests of vibration and thermal thresholds are normal, thus, supporting the suggestion that small fibre dysfunction precedes clinical neuropathy [10].

Previous cross-sectional studies of IENFD have shown a strong inverse relationship with age [11], [12], [13]. The purpose of this study was to determine whether the same is true for small fibre function assessed using the LDIflare technique and if so would the diagnostic accuracy for neuropathy detection be improved by the use of normative centiles.

## Methods

### 1. Ethics Statement

All subjects in the study gave written informed consent to participate in the study; ethical approval was obtained from the Essex 1 Ethics Committee. The study was carried out in accordance with the Declaration of Helsinki as revised in 2000.

### 2. Subjects

A total of 160 subjects were studied: 94 healthy subjects (52 F/44M) and 66 with diabetes. Healthy volunteers were recruited by advertisement and subjects with diabetes were recruited from those attending the Ipswich Diabetes Center. These included people with both type 1 and type 2 diabetes.

Healthy volunteers (HV) were enlisted to give approximately equal numbers in four age groups, <30 years, 30–45 years, 45–60 years and >60 years. Exclusion criteria, which also applied to those with diabetes, included current smoking, dysthyroid tests, known peripheral vascular disease or an abnormal ankle-brachial pressure index (ABPI), alcohol overuse, congestive heart failure, stroke, end-stage renal failure, uncontrolled hypertension or a history of cancer treatment. Subjects were also excluded if they had any current lesions on the foot. All healthy volunteers were free from clinical neuropathy assessed using two validated neuropathy scores - Neuropathy Disability Score (NDS) and the Toronto Clinical Neuropathy Score (TCNS). Glycaemic dsyregulation was excluded using a composite of fasting glucose <6.0 mmol/L and HbA1c <6.0% (42 mmol/mol).

Subjects with diabetes were divided into two groups; 1) those without clinical neuropathy (DN-); Neuropathy Disability Score [14] of less than 3, and Toronto Clinical Neuropathy Score of less than 5; and 2) those with clinical neuropathy (DN+); Neuropathy Disability Score ≥3, and Toronto Clinical Neuropathy Score ≥5) [15].

All participants were studied at a single institution, by the same investigators at each visit. Neuropathy scores were assessed by GR and LDIflares by PV. In those with diabetes, the examiner testing for LDIflare responses was unaware of the NDS or TCNS score until completion of the patient study.

### 3. Neuropathy Assessment

The Neuropathy Disability Score is a well validated tool to grade the severity of neuropathy based on objective clinical examination findings of qualitative vibration perception, temperature differentiation, pin prick sensation and presence of ankle reflexes [16]. No points are awarded for preserved sensation, but if impaired or absent 1 point is allocated per foot, except for ankle reflexes where 2 points are awarded if absent and 1 point if reflexes are present after distraction thus giving a cumulative maximum total of 10 points. A score of 0–2 was considered normal with scores of 3–5, 6–8, 9–10 indicating mild, moderate and severe neuropathy [8]. The Toronto Clinical Neuropathy Score (TCNS) is a grading system to evaluate history and physical examination components that permits stratification of clinical neuropathy into absent, mild, moderate and severe neuropathy and has been validated against morphological criteria of sural nerve fibre density [17]. Values of ≥5 signify the presence of clinical neuropathy.

### 4. Vibration Perception Thresholds Measurements (VPT)

All subjects had their VPT measured at the tip of the hallux with a Horwell Neurothesiometer using the method of limits. A minimum of 3 recordings were taken and their average determined.

### 5. Assessment of the LDIflare

The methodology for LDIflare technique is published in detail elsewhere [3], [18]. In the current study we used the recently modified technique that produces larger flares with greater reproducibility [18]. Briefly, after acclimatisation in a temperature-controlled room (25±1°C) and after the temperature of both the dorsum of the foot and the hallux exceeds 30°C a circular heating probe of 1 cm^2^ area is applied to the dorsum of the foot approximately 2–3 cm proximal to the first and second metatarsal heads. The skin is then heated for 6 min in a stepwise fashion starting at 44°C for 2 min, 46°C for 1 min and finally 47°C for 3 min using. After heating, the area is immediately scanned using an LDI (Moor Instruments, Axminster, UK). The flare area, identified on the computer as the area with hyperaemic response >300 perfusion units (PU) is measured using Moor V 5.3 software. The size of the LDIflare depends on C-fibre function and the underlying skin small fibre neural network and extent of interconnections.

### 6. Calculation of the Rate of Decline

The subject range was between 20 and 79 years, thus spanning six decades. The percentage difference between the 50^th^ centile of the youngest and oldest group was divided by a factor of 6, covering the six decades, to establish the percentage rate of decline per decade using the formula:

Percentage rate of decline in Flare area per decade:

*= [1−(Median flare area in Oldest group/Median Flare area in youngest group)×100]/Number of decades*.

This assumes a linear pattern of change which is apparent in the centile charts.

### Statistical Analysis

Data are presented as means ± SD. Variables were compared by ANOVA. Centile charts were determined in healthy volunteers using the simple empirical centiles method described by Wright and Royston [19]. Clinical neuropathy was diagnosed by a NDS of ≥3 and a TCNS of ≥5, from which the sensitivity and specificity of the LDIflare for detecting neuropathy, was determined by two different analytical methods:

1. Method A- That based on the age specific 5th centile values as the cut-off
2. Method B- That based on receiver operator characteristic curve (ROC) analysis which is a graphical plot of sensitivity on the Y axis against 1- Specificity [20]. Statistical analysis was performed using SPSS (version 17.0; SPSS,Chicago, IL).

## Results

Baseline characteristics of the two groups are detailed in Table 1.

**Table 1:** Baseline characteristics.

	HC	DN−	DN+	‘p’ for trend
Number of subjects	94	35	31	
Sex (F/M)	49/45	18/17	9/22	p = 0.046*
Age	44.5±16.4	54.1±9.0	56.8±14.6	p<0.01
DiabetesDuration (years)	0	7.6±7.5	8.0±4.5	p = 0.36
Diabetes (T1/T2)	0	20/15	11/20	
VPT (Volts)	6.9±3.8	10.1±1.4	22.7±10.7	p<0.001
NDS (max 10)	0.3±0.5	0.2±0.6	6.3±1.5	p<0.0001
TCNS (max 19)	0.4±1.0	0.7±1.0	7.3±2.6	p<0.0001
LDIflare (cm^2^)	9.2±2.9	6.9±2.7	2.7±0.9	p<0.0001

Values are means±SD.

* Chi-square test for categorical variable, rest ANOVA. HC = healthy controls, DN**−** = Diabetes without neuropathy and DN+ = Diabetes with neuropathy.

### 1. Healthy Volunteers

**Figure 1:**
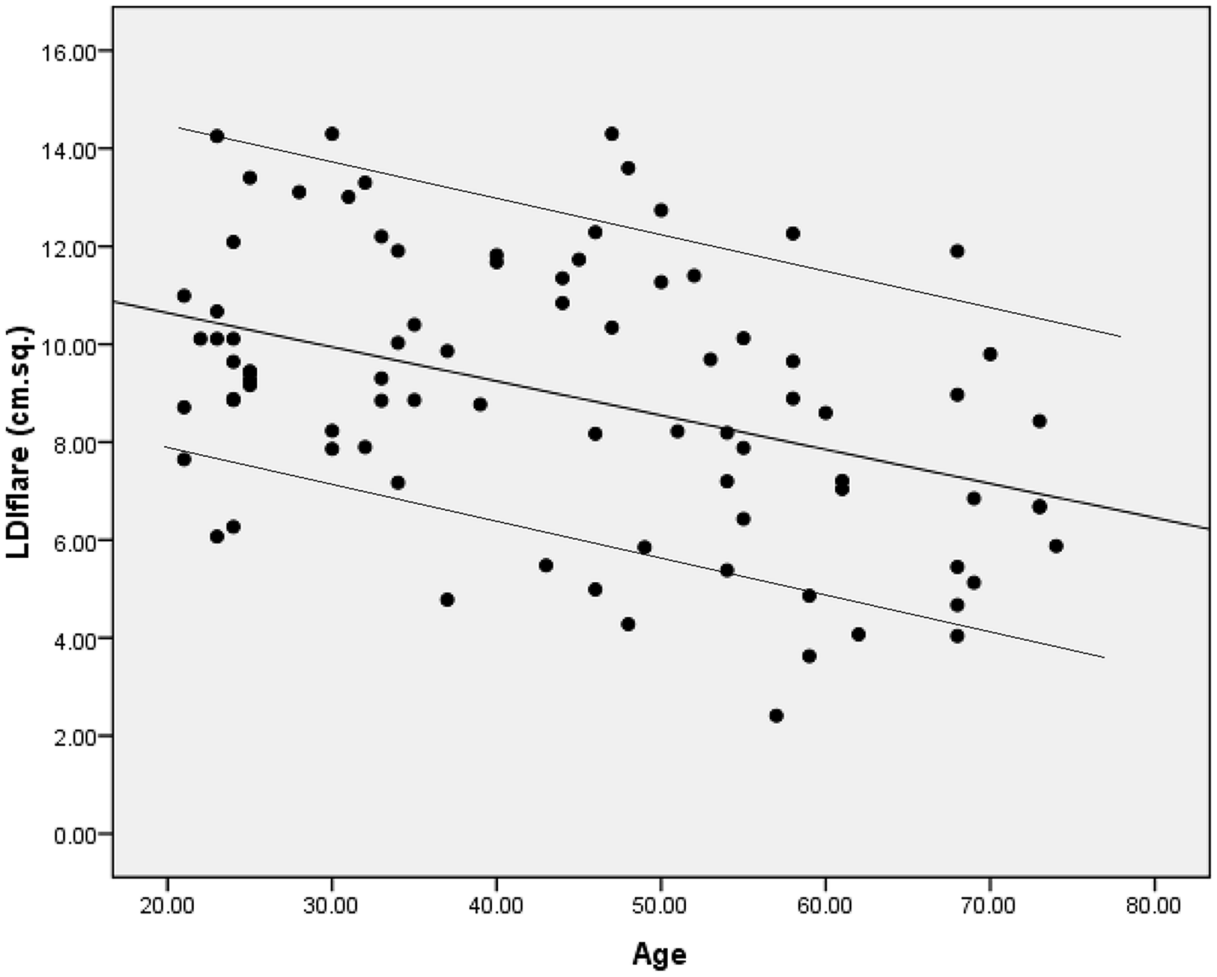
Relationship of the LDIflare (cm^2^) to age (years), r = −0.42, P<0.0001. **The lines above and below the trendline describe the 95^th^ and 5^th^ centiles respectively.**

There was a significant age dependant decrease in LDIflare size (r = −0.42, p<0.0001) with a rate of decline of 0.56 cm^2^ per decade giving a percentage loss of 5.5% per decade, Figure 1. Females had larger mean LDIflares than males (9.4±2.9 v 8.8±2.7 cm^2^), however this gender difference was not statistically significant (p = 0.22). The calculated 5th centiles for the youngest and oldest group were 8.4 cm^2^ and 4.1 cm^2^ respectively, Table 2. No relationship was observed with height, weight, BMI, VPT, HbA1C, or Total Cholesterol within the healthy volunteer group (not shown).

**Table 2:** Centile charts derived from 94 healthy controls divided into 4 age groups.

LDIflare (cm^2^)	Under 30 Years	30–45Years	45–60Years	Over 60Years
5th Centile	8.41	5.38	4.37	4.06
25^th^ Centile	9.27	8.79	6.28	5.56
50^th^ Centile	10.11	10.13	9.27	6.77
75^th^ Centile	13.11	11.79	11.48	8.13
95^th^ Centile	14.26	13.05	13.47	10.54

Centile charts derived from 94 healthy controls divided into 4 age groups.

### 2. Diabetes Groups

**Figure 2:**
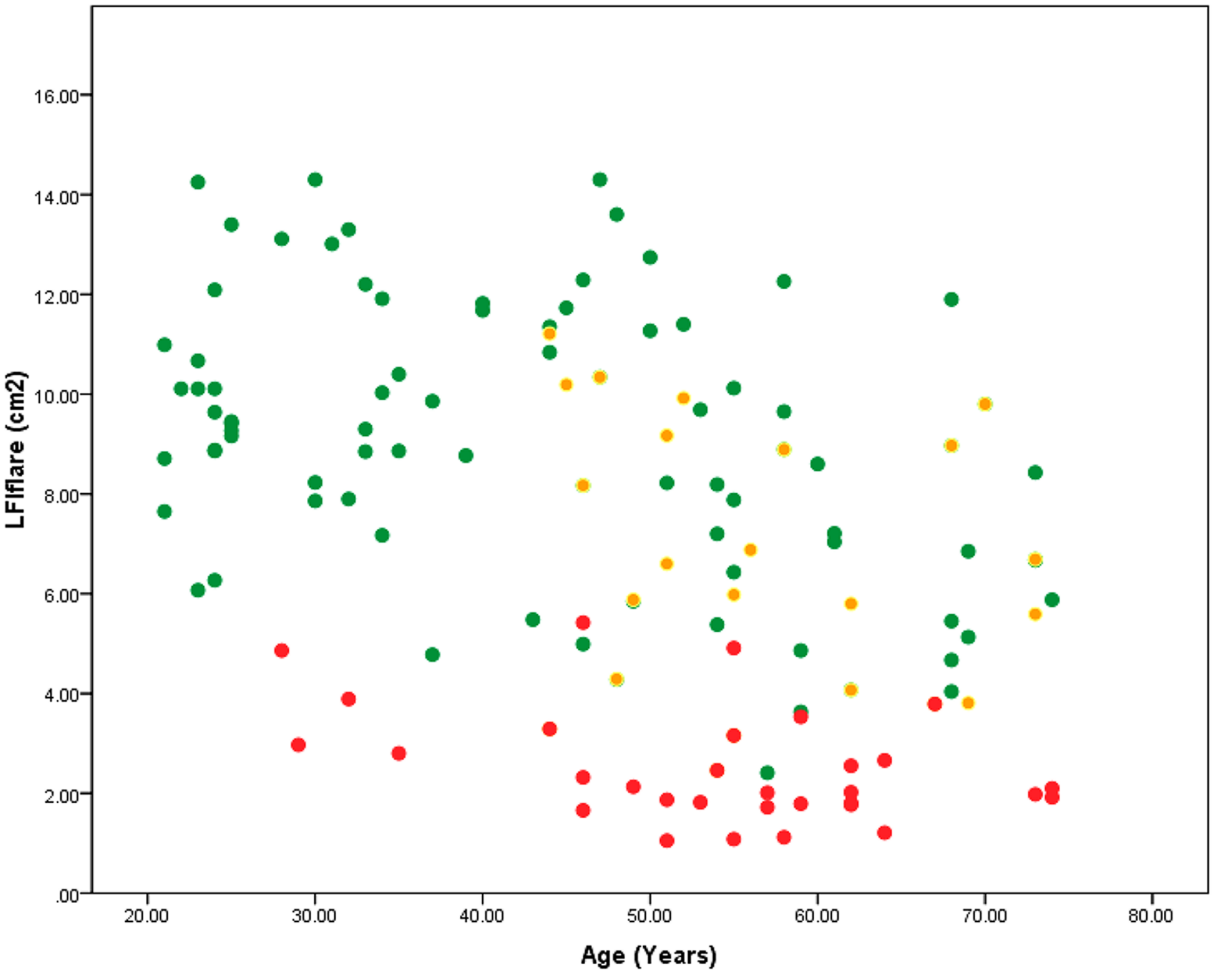
LDIflare relationship between the 3 groups. Green dots are the healthy controls (HC, flare area 9.2±2.9 cm^2^), amber dots are subjects with Diabetes but without clinical neuropathy (DN-, 6.9±2.79 cm^2^) and red dots the subjects with diabetes and clinical neuropathy (DN+, 2.7±0.9 cm^2^).

The DN+ group did not differ significantly from the DN- group in age (56.8±14.6 v 54.1±9.0 years, p = 0.19), or diabetes duration (8.00±4.51 v 7.55±7.47 percent, p = 0.77); however as expected vibration perception thresholds (22.7±10.7 v 10.1±1.4 Volts, p<0.001), Neuropathy Disability Score scores (6.33±1.5 v 0.67±0.9, p<0.001) and Toronto Clinical Neuropathy Score scores 7.32±2.61 v 0.66±0.98, p<0.0001) were significantly higher in the neuropathic group. The average ages of the diabetic groups were significantly greater than the HV group (54.1±9.0 for DN- and 56.8±14.6 for DN+, v 44.5±16.4 years, p<0.01). The LDIflare values were significantly lower in the DN+ group compared to DN- group (2.7±0.9 v 6.87±2.7 cm^2,^ p<0.0001), Figure 2.

### Sensitivities and Specificities Using the Two Methods

#### Method A

ROC analysis suggested an optimal cut-off threshold of 3.66 cm^2^ for the detection of clinical neuropathy, giving a sensitivity of 75% and specificity of 85%, with a Positive Predictive value (PPV) of 74% and a Negative Predictive value of (NPV) 86% (Table 3).

**Table 3:** Operating characteristics between the Method A and Method B.

	Sensitivity	Specificity	PPV	NPV
LDIflare with ROC threshold of <3.6 cm2	75%	85%	74%	86%
Using age-specificcut off values	77%	90%	82%	87%

PPV, Positive Predictive Value; NPV, Negative predictive value.

Operating characteristics between the Method A and Method B.

#### Method B

Using the 5% centile cut offs, the sensitivity was 77%, similar to that of method A but the specificity was better at 90%. The PPV was 82% and NPV 87%. (Table 3).

### Conclusions

This study demonstrates a significant age related reduction in small fibre function in the healthy population and is in keeping with findings of other studies of both small fibre structure [13], [21] and function [22]. The scatter plot of the individual results against age in our study is remarkably similar to those in two large studies for IENFD [12], [13]. Based on the tables given in these papers we are able to calculate the rate of loss of IENFD. From the worldwide normative reference study by Lauria, we calculate a fibre loss of approximately 0.9 fibres/mm per decade for combined sexes giving a 6 percent loss per decade. In the Umapathi study where fibre density was higher due to a different method used to calculate density, fibre loss was approximately 1.8 fibres/mm, but the percent decline was similar at 5.5 percent per decade. The reduction in LDIflare size of 0.56 cm^2^ per decade, is also remarkably similar at 5.5% decline per decade. Thus, it would appear that in healthy adults structural and functional loss parallel each other. However, the same may not be true in neuropathic states; further studies are required to determine this. Studying the rate of decline of nerve function against other biomarkers in disease states such as diabetes may help identify determinants of accelerated fibre loss. The advantage of the LDIflare technique over IENFD is that it is non invasive, causes no skin lesions and is therefore readily repeatable making it very suitable to examine rate of progression in longitudinal follow up studies.

Both analytic techniques demonstrate excellent operating characteristics for the LDIflare method in detecting individuals with clinical neuropathy, albeit the tests used to diagnose neuropathy in this study are largely based on large fibre abnormalities. Gibbons et al have determined the sensitivities and specificities of 26 different techniques used to diagnose neuropathy against the clinical diagnosis of neuropathy determined using the NDS score and analysed by the ROC method [23]. They found large variations in sensitivities and specificities, from 35 to 89% for the former and 39 to 78% for the latter. Tests with high sensitivities not infrequently had low specificities and vice versa. Overall, peroneal nerve amplitude performed best with a sensitivity of 73% and specificity of 78%. The LDIflare technique exceeds both these using either of the two analytical techniques we employed. However, the analysis based on age centiles showed greater specificity and hence a stronger positive predictive value. The use of the centile chart increases the diagnostic power for an individual and is readily apparent in the example of a twenty year old with a LDIflare value of 6 cm^2^, well above the ROC cut-off of 3.66 but well below the 5^th^ centile for age.

The sensitivities and specificities we report for the LDIflare method have to be considered in relation to the significantly lower results reported by Nabavi Nouri et al [24]. Using the ‘England’ as well as ‘sural nerve’ criteria for the diagnosis of neuropathy they reported sensitivities for the LDIflare technique of between 66% and 79% and specificities of between 60% and 70%. However, their mean LDIflare size for the normal group was only 3.4 cm^2^, considerably less than the 5.7 cm^2^ we reported for a similar age group using the same and earlier method we originally described [25]. It was also lower than the mean values we reported for older groups of normal subjects, in three different studies; 4.4 cm^2^, 5.2 cm^2^ [3], [10] and 5.2 cm^2^ [26]. The reliability of a test will depend on its reproducibility. In our studies this varied between 11–13% (coefficient of variation). We are unaware of reproducibility figures from the above investigators. It is therefore possible that the less favourable operating characteristics may be down to technical differences in the application of the method. Furthermore, it should be noted that in the current study we have used a newer method employing a higher temperature. This gives consistently larger flares than the previous method. Finally, Ebadi et al, from the same unit, studied patients with symptoms suggestive of small fibre neuropathy, arbitrary dividing them into a small fibre sensory neuropathic (SFSN) group based on an IENFD of below 5.4 fibres/mm and a ‘normal’ but symptomatic group in which the IENFD was above 5.4 fibres/mm [27]. The LDIflares in the ‘normal’ symptomatic group were not significantly different (2.3±1.2 v 2.1±1.1 cm^2^) from those in the SFSN group with a resulting sensitivity of only 54% and a specificity of 54% in detecting small fibre neuropathy. However, compared with mean of the true healthy volunteers (3.4±1.9 cm^2^) in the previously mentioned Nabavi Nour paper, both groups have very small LDIflare results suggesting that both have small fibre dysfunction. Thus, the so called ‘normal’ group’s neuropathic symptoms may be the result of small fibre dysfunctional in the presence of normal small fibre density. It may therefore, be misleading to derive the sensitivities and specificities of a test for detection of small fibre neuropathy based solely on INEFD. Indeed if based on symptoms only the LDIflare may have revealed very high operating characteristics.

To date corneal confocal microscopy studies have shown good sensitivities (range 60–91%) and specificities (range 45%–79%) in detecting clinical neuropathy (NDS ≥3) using the ROC analytical method, and also progressive reduction in corneal fibre structural features with progressive neuropathy [8]. The use of centile based analysis may also improve the operating characteristics of corneal confocal microscopy in detecting neuropathy.

A limitation of this study is using composite clinical scores to diagnose neuropathy as these that mainly relate to large fibre neuropathy. It could be argued that it would have been useful to have performed quantitative sensory testing as studies have shown it to be a objective measure [28], [29] but in our previous studies we found these time consuming and not as helpful as expected, perhaps due to their subjective nature [6]. Ideally, it would have been useful to have included electrophysiological assessments, corneal confocal microscopy and IENFD. We would encourage future studies to include all such methodologies.

In summary, we have demonstrated for the first time, a decline of nerve function in a normal population and have also derived normative charts. We have also shown that the use of such centile charts improves the sensitivities and specificities for the diagnosis of clinical neuropathy. To investigate the aetiopathogenesis of various neuropathies requires a test that ideally detects its earliest stages, is non invasive, quantifiable, can follow temporal changes in response to changes in the disease process, and can be potentially linked pathogenetic biomarkers and therapeutic interventions. Furthermore, it needs to have high sensitivity, specificity and reproducibility. We believe the LDIflare has these attributes.

## References

[1] Boulton AJ, Malik RA (2010) Neuropathy of impaired glucose tolerance and its measurement. Diabetes Care 33: 207–209. 20040677 10.2337/dc09-1728 PMC2797976

[2] Smith AG, Russell J, Feldman EL, Goldstein J, Peltier A, et al (2006) Lifestyle intervention for pre-diabetic neuropathy. Diabetes Care 29: 1294–1299. 16732011 10.2337/dc06-0224

[3] Krishnan ST, Rayman G (2004) The LDIflare: a novel test of C-fiber function demonstrates early neuropathy in type 2 diabetes. Diabetes Care 27: 2930–2935. 15562209 10.2337/diacare.27.12.2930

[4] Tavakoli M, Marshall A, Pitceathly R, Fadavi H, Gow D, et al.. (2009) Corneal confocal microscopy: A novel means to detect nerve fibre damage in idiopathic small fibre neuropathy. Exp Neurol. 10.1016/j.expneurol.2009.08.033 PMC2938826 19748505

[5] Maser RE, Nielsen VK, Bass EB, Manjoo Q, Dorman JS, et al (1989) Measuring diabetic neuropathy. Assessment and comparison of clinical examination and quantitative sensory testing. Diabetes Care 12: 270–275. 2707114 10.2337/diacare.12.4.270

[6] Shy ME, Frohman EM, So YT, Arezzo JC, Cornblath DR, et al (2003) Quantitative sensory testing: report of the Therapeutics and Technology Assessment Subcommittee of the American Academy of Neurology. Neurology 60: 898–904. 12654951 10.1212/01.wnl.0000058546.16985.11

[7] Freeman R, Chase KP, Risk MR (2003) Quantitative sensory testing cannot differentiate simulated sensory loss from sensory neuropathy. Neurology 60: 465–470. 12578928 10.1212/wnl.60.3.465

[8] Tavakoli M, Quattrini C, Abbott C, Kallinikos P, Marshall A, et al (2010) Corneal confocal microscopy: a novel noninvasive test to diagnose and stratify the severity of human diabetic neuropathy. Diabetes Care 33: 1792–1797. 20435796 10.2337/dc10-0253 PMC2909064

[9] Green AQ (2010) Small fibre function assessed using the LDI flare technique in subjects with type 1 diabetes mellitus and impaired glucose tolerance. 146.

[10] Krishnan ST, Quattrini C, Jeziorska M, Malik RA, Rayman G (2009) Abnormal LDIflare but normal quantitative sensory testing and dermal nerve fiber density in patients with painful diabetic neuropathy. Diabetes Care 32: 451–455. 19074993 10.2337/dc08-1453 PMC2646027

[11] McArthur JC, Stocks EA, Hauer P, Cornblath DR, Griffin JW (1998) Epidermal nerve fiber density: normative reference range and diagnostic efficiency. Arch Neurol 55: 1513–1520. 9865794 10.1001/archneur.55.12.1513

[12] Umapathi T, Tan WL, Loke SC, Soon PC, Tavintharan S, et al (2007) Intraepidermal nerve fiber density as a marker of early diabetic neuropathy. Muscle Nerve 35: 591–598. 17221881 10.1002/mus.20732

[13] Lauria G, Bakkers M, Schmitz C, Lombardi R, Penza P, et al (2010) Intraepidermal nerve fiber density at the distal leg: a worldwide normative reference study. J Peripher Nerv Syst 15: 202–207. 21040142 10.1111/j.1529-8027.2010.00271.x

[14] Boulton AJM (2005) Management of Diabetic Peripheral Neuropathy. Clinical Diabetes 23: 9–15.

[15] Bril V, Perkins BA (2002) Validation of the Toronto Clinical Scoring System for Diabetic Polyneuropathy. Diabetes Care 25: 2048–2052. 12401755 10.2337/diacare.25.11.2048

[16] Abbott CA, Carrington AL, Ashe H, Bath S, Every LC, et al (2002) The North-West Diabetes Foot Care Study: incidence of, and risk factors for, new diabetic foot ulceration in a community-based patient cohort. Diabet Med 19: 377–384. 12027925 10.1046/j.1464-5491.2002.00698.x

[17] Bril V, Perkins BA (2002) Validation of the Toronto Clinical Scoring System for diabetic polyneuropathy. Diabetes Care 25: 2048–2052. 12401755 10.2337/diacare.25.11.2048

[18] Vas PR, Rayman G (2012) Validation of the modified LDIFLARE technique: A simple and quick method to assess C-fiber function. Muscle Nerve. 10.1002/mus.23532 23169592

[19] Wright EM, Royston P (1999) Calculating reference intervals for laboratory measurements. Statistical Methods in Medical Research 8: 93–112. 10501648 10.1177/096228029900800202

[20] Bewick V, Cheek L, Ball J (2004) Statistics review 13: Receiver operating characteristic curves. Critical Care 8: 508–512. 15566624 10.1186/cc3000 PMC1065080

[21] Bakkers M, Merkies IS, Lauria G, Devigili G, Penza P, et al (2009) Intraepidermal nerve fiber density and its application in sarcoidosis. Neurology 73: 1142–1148. 19805731 10.1212/WNL.0b013e3181bacf05

[22] Bravenboer B, van Dam PS, Hop J, v.d. Steenhoven J, Erkelens DW (1992) Thermal Threshold Testing for the Assessment of Small Fibre Dysfunction: Normal Values and Reproducibility. Diabetic Medicine 9: 546–549. 1643803 10.1111/j.1464-5491.1992.tb01836.x

[23] Gibbons CH, Freeman R, Veves A (2010) Diabetic neuropathy: a cross-sectional study of the relationships among tests of neurophysiology. Diabetes Care 33: 2629–2634. 20805259 10.2337/dc10-0763 PMC2992203

[24] Nabavi Nouri M, Ahmed A, Bril V, Orszag A, Ng E, et al (2012) Diabetic neuropathy and axon reflex-mediated neurogenic vasodilatation in type 1 diabetes. PLoS One 7: e34807. 22529938 10.1371/journal.pone.0034807 PMC3328500

[25] Green AQ, Krishnan ST, Rayman G (2009) C-fiber function assessed by the laser doppler imager flare technique and acetylcholine iontophoresis. Muscle Nerve 40: 985–991. 19768772 10.1002/mus.21333

[26] Green AQ, Krishnan S, Finucane FM, Rayman G (2010) Altered C-fiber function as an indicator of early peripheral neuropathy in individuals with impaired glucose tolerance. Diabetes Care 33: 174–176. 20040675 10.2337/dc09-0101 PMC2797968

[27] Ebadi H, Perkins BA, Katzberg HD, Lovblom LE, Bril V (2012) Evaluation of proxy tests for SFSN: evidence for mixed small and large fiber dysfunction. PLoS One 7: e42208. 22870304 10.1371/journal.pone.0042208 PMC3411719

[28] Rolke R, Baron R, Maier C, Tolle TR, Treede RD, et al (2006) Quantitative sensory testing in the German Research Network on Neuropathic Pain (DFNS): standardized protocol and reference values. Pain 123: 231–243. 16697110 10.1016/j.pain.2006.01.041

[29] Blankenburg M, Boekens H, Hechler T, Maier C, Krumova E, et al (2010) Reference values for quantitative sensory testing in children and adolescents: developmental and gender differences of somatosensory perception. Pain 149: 76–88. 20138430 10.1016/j.pain.2010.01.011

